# In situ temperature measurements in sooting methane/air flames using synchrotron x-ray fluorescence of seeded krypton atoms

**DOI:** 10.1126/sciadv.abm7947

**Published:** 2022-04-29

**Authors:** Matthew J. Montgomery, Hyunguk Kwon, Alan L. Kastengren, Lisa D. Pfefferle, Travis Sikes, Robert S. Tranter, Yuan Xuan, Charles S. McEnally

**Affiliations:** 1 Department of Chemical and Environmental Engineering, Yale University, New Haven, CT, USA.; 2 Department of Chemical Engineering, Pennsylvania State University, University Park, PA, USA.; 3 X-Ray Science Division, Argonne National Laboratory, Argonne, IL, USA.; 4 Chemical Sciences and Engineering Division, Argonne National Laboratory, Lemont, IL, USA.; 5 Department of Mechanical Engineering, Pennsylvania State University, University Park, PA, USA.

## Abstract

Synchrotron x-ray fluorescence has been used to measure temperatures in optically dense gases where traditional methods would fail. These data provide a benchmark for stringent tests of computational fluid dynamics models for complex systems where physical and chemical processes are intimately linked. The experiments measured krypton number densities in a sooting, atmospheric pressure, nonpremixed coflow flame that is widely used in combustion research. The experiments not only form targets for the models, but the simulations also identify potential sources of uncertainties in the measurements, allowing for future improvements.

## Teaser

State-of-the-art x-ray fluorescence methods overcome issues in measuring gas temperatures that hinder traditional diagnostics.

## INTRODUCTION

X-ray techniques (XRTs) have been used extensively to characterize solid samples and have contributed to many Nobel Prizes awarded in chemistry, physics, and medicine (*1*), but x-ray measurements in gas-phase environments are much less common. This gap is unfortunate because gas-phase reacting flows—such as the flames used in most engines and other energy-generating systems—are an important topic of research to improve efficiency and reduce emissions (*2*). The challenge is that the material density in flames is orders of magnitude lower than in solids, especially because the temperature can exceed 2000 K. However, these measurements are becoming feasible because of the greater availability of synchrotron x-ray sources with high brilliance (*3*) and improvements in x-ray optics, such as Kumakhov lenses, which can efficiently collect emitted or scattered x-rays over an extended solid angle (*4*). Several recent studies have used XRTs to measure temperature or species concentrations in flames (*5*–*10*). For example, x-ray computed tomography has been used to image premixed methane/air flames composed of 25 to 65% krypton by mole fraction, and temperatures were obtained from x-ray attenuation measurements (*10*). In this study, x-ray fluorescence (XRF) was used to measure temperatures throughout a methane/air flame seeded with only 2.4% krypton by mole fraction. These data were used to rigorously validate a detailed computational fluid dynamics (CFD) simulation of the flame.

This work exploits the unique properties of x-rays to overcome three issues with the ultraviolet/visible (UV/VIS) laser techniques (UVTs) conventionally used in combustion research. First, it demonstrates that XRTs are unaffected by complications from carbonaceous soot particles. Most flames produce substantial quantities of soot; indeed, the yellow color universally associated with fire is due to blackbody radiation from heated soot particles (*11*). Emissions of these particles have severe consequences: They contribute to ambient fine particulates that cause millions of annual deaths (*12*), and they are the largest source of global warming after CO_2_ (*13*). Unfortunately, soot particles interfere with UVT measurements because of their strong luminosity, scattering, and absorption at the relevant wavelengths [e.g., (*14*–*16*)], which limits research to understand and reduce their formation. However, soot particles in flames do not emit blackbody radiation in the x-ray regime. Scattering from particles is also greatly reduced because the index of refraction of all materials is the same to within ~10 parts per million at x-ray wavelengths (*17*–*19*). Last, all light atoms, including carbon, negligibly absorb hard x-rays over the length scales of flames (*18*, *20*).

A second benefit of XRT is that they depend much less than UVT on ambient conditions in the measurement region. For example, at atmospheric pressure and above, the primary removal mechanism for excited electronic states is collisional quenching; therefore, fluorescence signals in UVT are strong functions of the bath gas composition, such that their interpretation requires detailed information on energy transfer rate constants and independent measurements of species concentrations (*21*). In contrast, the primary removal mechanism for the core-hole ions probed with the XRF technique in this study is internal rearrangement to more stable ions, with a lifetime of 0.17 fs (*22*), which is orders of magnitude shorter than the collisional period. Similarly, thermal energies, even at flame temperatures, are negligible (~0.2 eV) compared to hard x-ray energies (>6000 eV), so the parameters involved in XRT are insensitive to temperature. In this study, temperature is recovered entirely from measured number density via the ideal gas law, and no temperature corrections are necessary.

Third, XRTs are ideal for measuring properties near the surfaces of a burner. CFD simulations are essential to optimize combustion systems (*23*), but they rely on accurate specification of the boundary conditions at the edges of the computational domain (*24*). XRT measurements can directly interrogate these conditions because x-rays do not reflect from surfaces except at shallow grazing incidence angles (<1°). Furthermore, the diameter of a focused radiation beam scales with its wavelength, so x-rays can be focused to a narrower diameter [~6 mm in our case; (*25*)] than UV/VIS beams. Last, XRT can make measurements inside opaque sections of a burner if they are made of low–atomic number materials such as SiC or Al (*26*).

The flame studied in this work was generated with a coflow nonpremixed burner (fig. S1) that has been chosen as a canonical configuration by the International Sooting Flame Workshop, a global research collaboration intended to improve CFD simulations of soot formation (*27*). Hence, the results of this work can be related to the work of numerous other research groups (*28*–*33*). The specific methane/air flame in this study is one that we have used extensively to study soot formation from advanced biofuels (*34*–*37*).

The XRF technique used in this study used the same procedures as Kastengren *et al.* (*6*, *9*). Krypton was seeded as an inert tracer at 2.3% into the reactant streams. A monochromatic x-ray beam at 15.1-keV mean photon energy excited the krypton atoms to a core-hole ion, and the resulting Kα x-ray fluorescence at 12.6 keV was detected. A polycapillary x-ray optic was used to limit the probe volume size to 4 μm by 6 μm by 320 μm full width at half maximum (FWHM). This fluorescence was well separated from other spectral features (fig. S2), including other Kr emission lines (*38*), elastic scattering, Compton scattering, and potential emission lines from C, H, O, and N. The fluorescence signal is directly proportional to Kr number density and can be calibrated from measurements in the unreacted gases where the Kr concentration is known. The pressure and Kr mole fraction are expected to be virtually constant throughout the flow because the burner is open to the atmosphere, and Kr is inert, so temperature can be determined from the Kr number density via the ideal gas law. It should be noted that this technique relies on the assumption that the krypton mole fraction is constant throughout the flow, which is not strictly true for fuels where moles are not conserved upon combustion (e.g. hydrogen) or in combustion regions containing intermediate species. Computational simulations were used in the current study to verify that the errors in this assumption are small, and these predictive methods can be extended to estimate the uncertainties in these assumptions for other fuels such as hydrogen. The measurements were performed using Beamline 7-BM at the Advanced Photon Source (APS) (*25*); the high brilliance of this synchrotron source allowed the Kr doping level to be small enough to not perturb the flame.

## RESULTS AND DISCUSSION

**Fig. 1.**
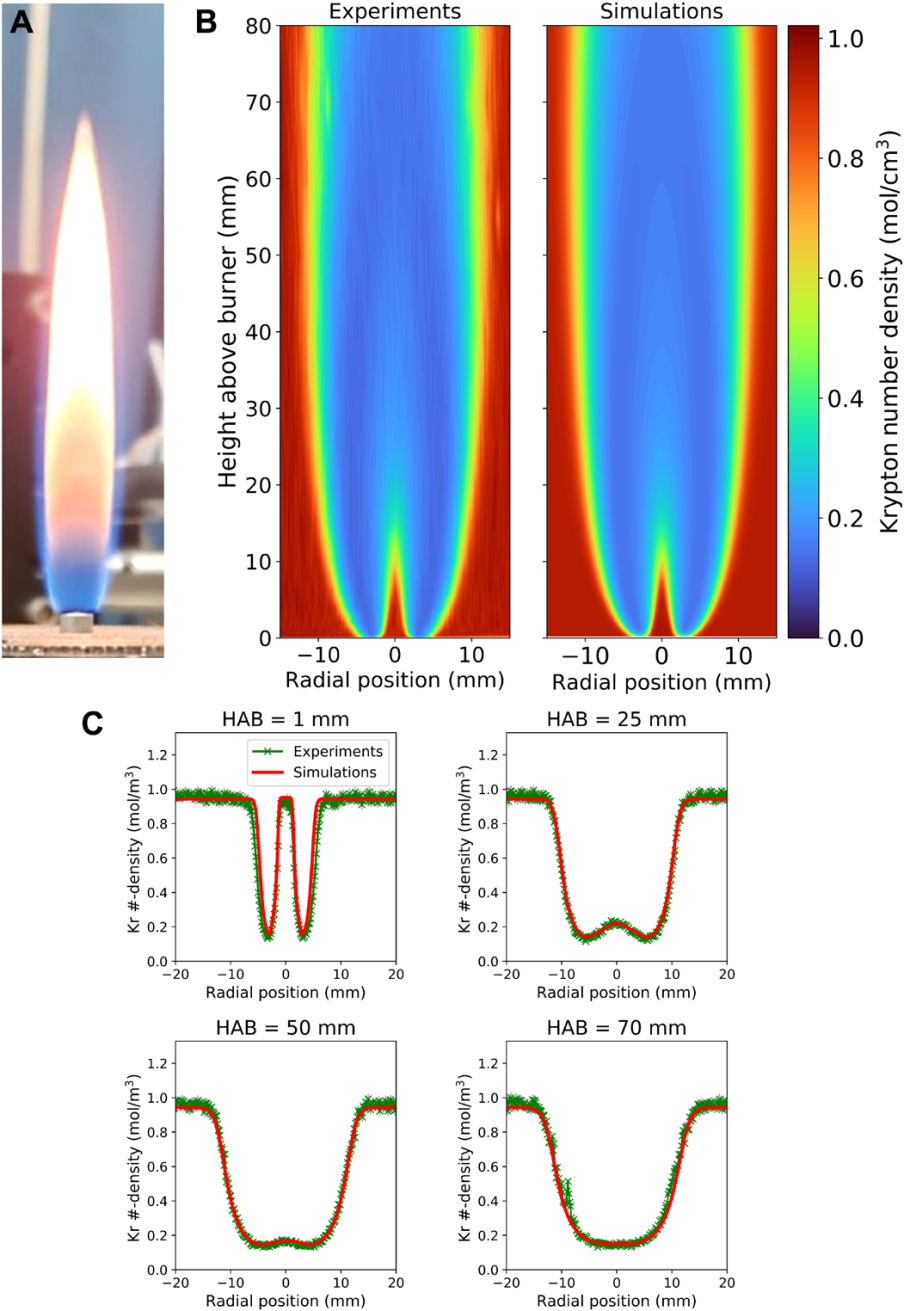
Measured and simulated krypton number densities in a sooting methane/air flame. (**A**) A photograph of the flame sized to the same spatial scale as (B). (**B**) Image plots of experimental (left) and simulated (right) krypton number densities throughout the flame. (**C**) Radial profiles of krypton number density at several heights above burner (HABs). Error bars for the measurements are represented by gray-shaded regions. Total time to collect 2D data: 2 hours.

**Fig. 2.**
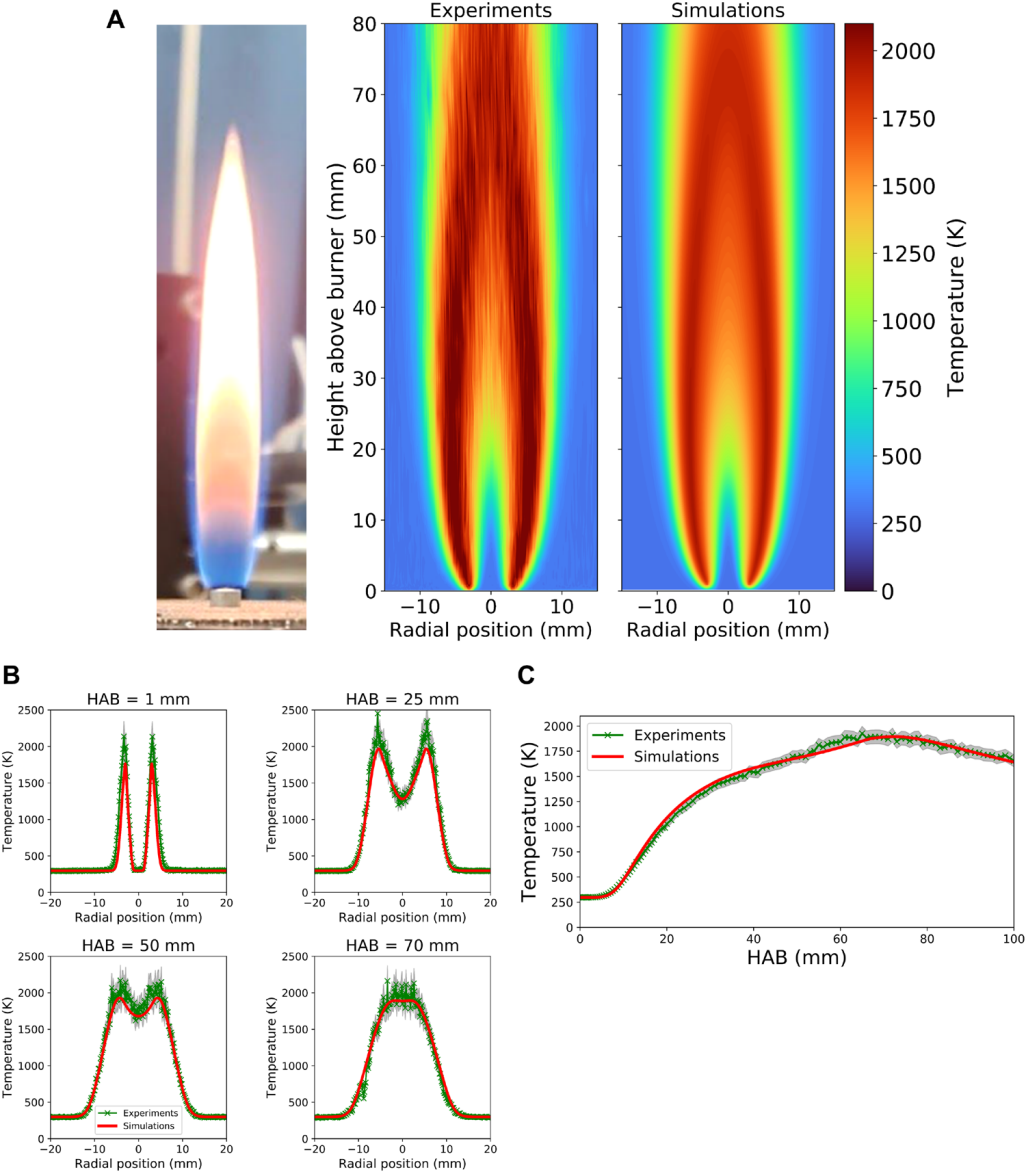
Measured and simulated gas temperatures calculated from the krypton number densities in Fig. 1 via the ideal gas law. (**A**) Image plots of experimental (left) and simulated (right) gas temperatures throughout the flame. (**B**) Radial profiles of gas temperatures at several HABs. (**C**) Centerline temperatures in the flame. The detection time for these measurements was 20 s per data point. For (B) and (C), error bars for the measurements are represented by the gray-shaded regions. Total time to collect 2D data: 2 hours. Total time to collect 1D data (C): 42 min.

The experimentally measured krypton number densities are displayed in Fig. 1, along with the simulated profiles. A photograph of the flame is shown in Fig. 1A. The experimental and simulated temperatures are shown in Fig. 2. Experimental and simulated radial plots at various heights above burner (HABs) are also shown in each figure. The experimental two-dimensional (2D) profiles (Figs. 1B and 2A) show that the technique was able to successfully capture the shape of the flame and had sufficient resolution to resolve sharp gradients in krypton number densities and temperatures. The experimentally measured profiles show outstanding agreement with the simulated results, demonstrating that the XRF technique was successful in obtaining temperatures in both sooting and nonsooting regions of the flame. It should also be noted that gas temperatures were obtained at HAB values as low as 100 μm. The radial plots (Figs. 1C and 2B) reveal that the largest differences between the experimental and simulated values occur along the wings of the flame and toward the centerline at higher HAB values. This observation is due to the minor variations in the krypton mole fraction (fig. S3) and the high temperatures encountered in these regions of the flame, which results in a lower collected fluorescent signal and consequently larger uncertainty from photon shot noise (fig. S4).

Experimental and simulated centerline temperature profiles are shown in Fig. 2C. For these measurements, the collection time was increased from 1 to 20 s per data point. The centerline temperatures, as well as 2D temperature profiles, reveal that the gases along the centerline remain at room temperature until reactions begin to occur at ~10-mm HAB. The temperature then begins to increase at this point along the centerline until the temperature peaks between ~60 and 70 mm HAB. This trend is also accurately predicted by the simulations. The predicted peak centerline temperature also matches well with the measured value, agreeing within <1.8%. It should be noted that above HAB = 55 mm, minor noise is visible in the measured temperatures. This is attributable to uncertainty introduced by unavoidable temperature fluctuations/flickering in the flame at these regions and could be better resolved with a longer detection time. However, within the uncertainty in the experiments, the measured and simulated centerline temperatures are in remarkable agreement.

**Fig. 3.**
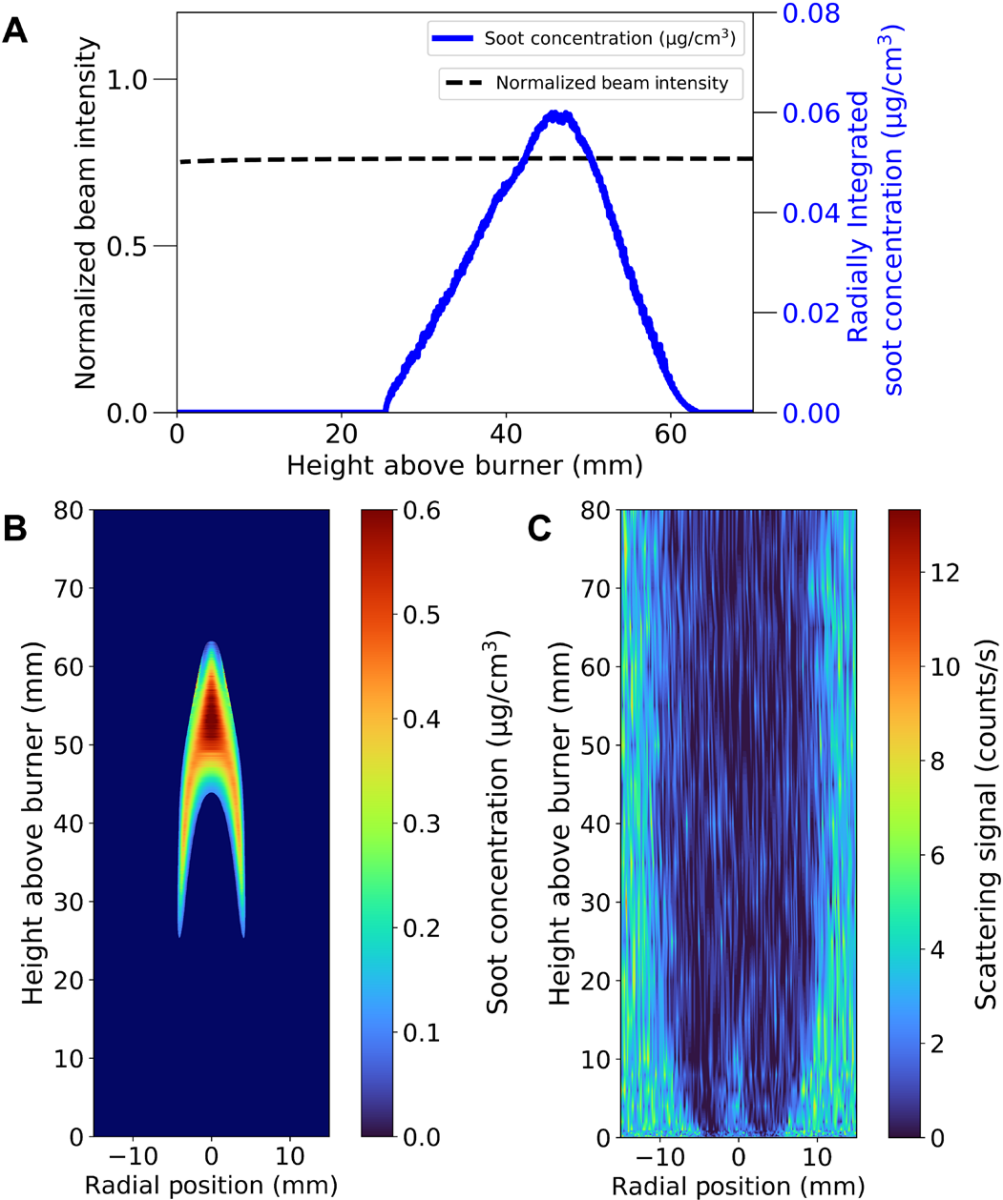
Dependence of the experimental gas-phase measurements on the presence of soot particles. (**A**) Radially integrated soot volume fraction and transmitted beam intensity versus HAB. (**B**) 2D soot concentrations from (*58*), for the same CH_4_ flame in this study except without seeded krypton atoms. (**C**) Scattering profile in the Kr-seeded CH_4_ flame.

To provide evidence that soot does not interfere with the experimental measurement, we determined the transmitted beam intensity, normalized to the incident beam intensity, and the radially integrated soot volume fraction profiles along the height of the flame and displayed them in Fig. 3A. There was no notable difference in the transmitted beam intensity along the centerline despite the soot volume fraction changing as the HAB varied. Given a constant incident beam intensity, these results suggest that soot does not interfere with the absorption of incident 15-keV photons. In Fig. 3B, a plot showing the 2D soot concentrations in the flame is shown, along with the 2D scattering signal in the flame for comparison in Fig. 3C. The scattering signal includes elastic and inelastic scattering contributions detected from 14.6 to 15.2 keV. The scattering signal is lowest toward the centerline of the flame but gets larger toward the oxidizer side of the flame. The major contribution to the scattering signal is expected to be the Compton scattering, which itself is proportional to the electron density and, hence, temperature inside the flame (*5*). Thus, the larger scattering signal in the oxidizer region relative to the fuel is explained by the larger electron density found in the colder oxidizer region compared to the flame. By comparing the 2D soot concentrations to the scattering profile, it can be seen that the scattering signal inside the flame is unaffected by the presence of soot. On the basis of these results, it can be concluded that the measurement is free from interference due to soot in these nonpremixed CH_4_ flames, and the measured signal is well separated from other spectral interferences (fig. S2).

The main uncertainties governing the accuracy of the temperature measurements are due to (i) the assumption that the krypton mole fraction is constant throughout the flame and (ii) the acquisition time for collecting the data. Variations in pressure were considered negligible relative to other sources of uncertainty (fig. S5). On the basis of the uncertainty introduced by variations in *X*_Kr_ (fig. S3) and random uncertainty from photon shot noise (fig. S4), we estimate a minimum (maximum) uncertainty of ±4.5% (±10.2%) in measured 2D temperatures (fig. S6). The uncertainty in the centerline measurements is ≤3.6%. It should be noted that the photon collection time for the 2D measurements was 1 s, and maximum uncertainties less than 5% can be achieved by increasing the photon collection time to 6 s because the photon shot noise scales with $1/\sqrt{n}$ (*39*).

In combustion modeling of laminar flames, thermal boundary conditions are required as inputs and have been assumed to be ambient temperature in past cases (*24*, *40*, *41*) due to experimental difficulties in carrying out these measurements. Recent boundary condition thermometry measurements in diffusion flames using coflow burners such as the one in this study, however, suggest that this may not be an accurate assumption (*42*). Accurately capturing thermal boundary conditions is important because uncertainties in these initial conditions can influence modeling results, such as predictions for peak flame temperature, flame shape, and emissions such as soot concentrations (*24*, *43*).

The XRF technique was able to measure flame temperatures as low as 100 μm above the burner surface. Previous studies have found that thermal boundary conditions measured with a thermocouple at an HAB of 100 μm strongly correlate with boundary conditions determined at the burner surface with phosphor thermometry (*42*). On this basis, it seems reasonable to conclude that the temperatures measured with the XRF technique at an HAB of 100 μm are correlated with those found at or near the burner surface. Likewise, temperatures predicted by the simulations at these near-burner HABs would be expected to depend strongly on the thermal boundary conditions. By comparing the near-burner temperatures determined with XRF to the predicted values, one can gauge the accuracy of the thermal boundary conditions input to the simulations.

**Fig. 4.**
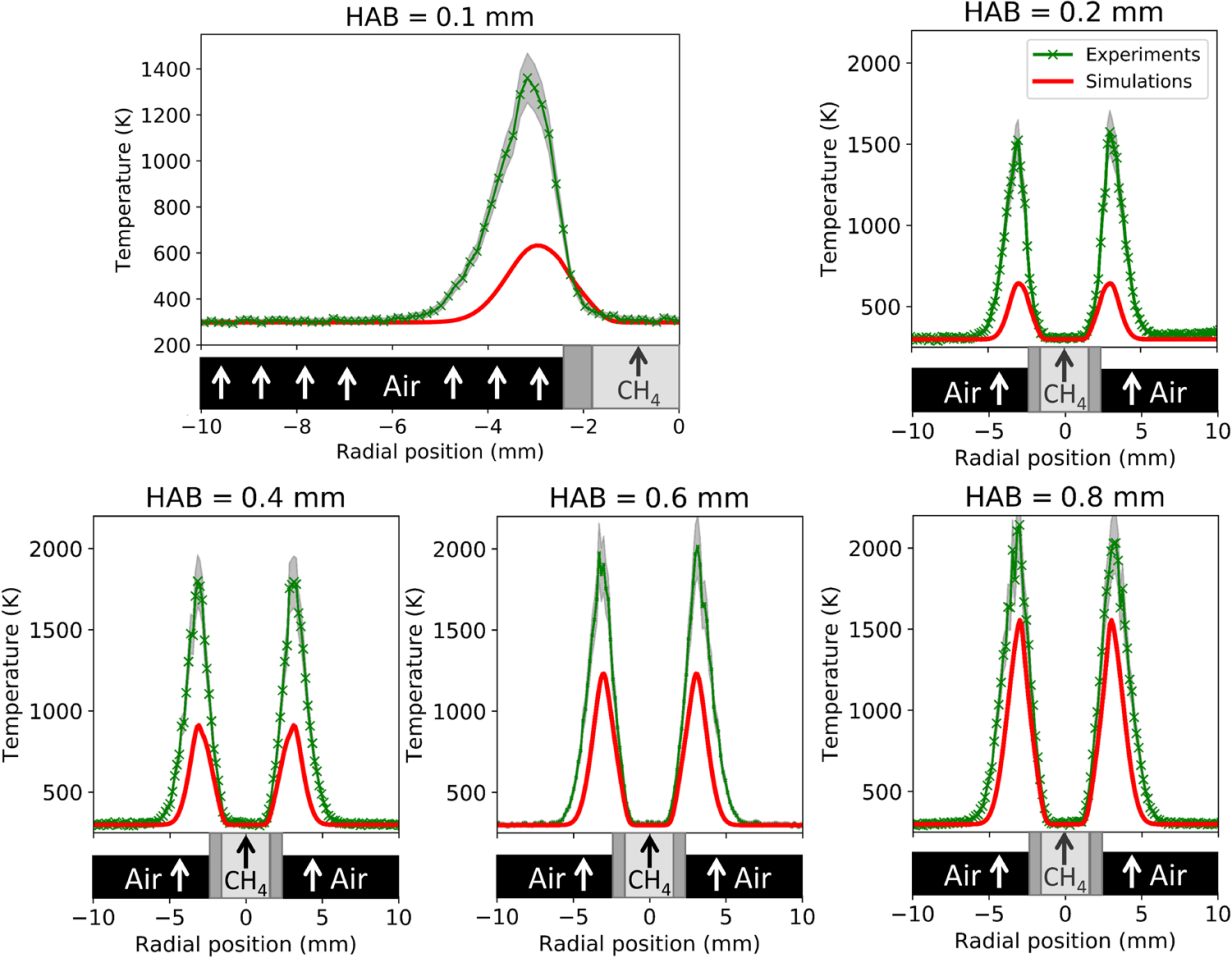
Radial profiles of near-burner temperatures, from HAB = 0.1 to 0.8 mm. Depictions of the burner/fuel tube are shown to approximate scale in the radial direction. Error bars for the measurements are represented by the gray-shaded regions.

Radial plots of the near-burner temperatures measured with this technique are compared to the simulated temperatures in Fig. 4. For this study, it should be noted that the thermal boundary conditions were taken as *T* = 298 K in both the fuel and oxidizer. The measured and simulated profiles both show that the temperature peaks in the wings of the flames at a radial position of ±3 mm and increases rapidly as the HAB increases. The measured temperatures agree well with the predicted temperatures in the farther coflow regions and right above the fuel tube and reveal that the areas above the edge of the fuel tube are not at room temperature. The measured temperatures in the wings of the flame were found to be >100 K higher than those predicted by the simulations at the examined near-burner temperatures, which in part is attributable to experimental uncertainty. However, the large differences that are evident at low HAB may suggest that the thermal boundary conditions are not accurately captured and require further experimental investigation to measure correctly. Despite the disagreement observed in near-burner temperatures caused by unrealistic boundary conditions input to the simulations, the predicted and measured temperatures quickly converge at higher HAB (Fig. 2, B and C), and the overall flame shapes are well matched.

In this work, the accuracy of 2D-resolved synchrotron XRF for measuring temperatures in sooting flames has been established. The technique was found to be insensitive to the presence of soot particles and was used to measure krypton number densities and temperatures in both nonsooting and sooting regions of the flame. The technique was also able to resolve sharp gradients in these variables over a wide dynamic range. The simulated krypton number densities and temperatures were in overall great agreement with the measured values. Near-burner temperatures were also obtained and suggest that temperatures at the burner surface are not at ambient conditions, as are typically assumed in simulations, including those presented here. The largest disagreements were observed in the wings of the flames near the burner surface, which is attributed to inaccurate boundary conditions input to the simulations. For future measurements, the photon collection time could be increased to 6 s to limit the uncertainty to ≤5%. While demonstrated as a combustion diagnostic, the technique presented here is expected to provide advantages for measuring temperatures in other gas-phase systems, e.g., in the vicinity of a catalyst’s surface during a reaction, during gas-phase syntheses, and during aerosol formation.

## MATERIALS AND METHODS

### Strategy for temperature measurements

Temperature was measured via the following steps: (i) Krypton was doped into both reactant streams of the flame at an initial mole fraction *X*_Kr,0_ = 0.023; (ii) the krypton number density *n*_Kr_ was measured throughout the flame by XRF; (iii) the temperature *T* was determined by the ideal gas law and the assumption that *X*_Kr_ = *X*_Kr,0_ throughout the flame; (iv) the measured *T* was compared to simulated *T*; and (v) the validated simulation was used to estimate the uncertainties in *T* due to the assumption that *X*_Kr_ = *X*_Kr,0_.

XRF, as implemented in this study, is a two-step process. First, a 15-keV photon from the incident x-ray beam is absorbed by a krypton atom and ionizes it to a core-hole ion

$$P_{15\text{keV}}+\text{Kr}(1s^{2}\;2s^{2}\;2p^{6}\ldots 4p^{6})\to {\text{Kr}}^{+}(1s^{1*}\;2s^{2}\;2p^{6}\ldots 4p^{6})+e^{–}$$

The ionization predominantly occurs at the 1s electrons, and not the valence 4p electrons, because the incident photon energy slightly exceeds the binding energy for the 1s electrons (14.3 keV) (*44*). The core-hole ion that is formed predominantly stabilizes by relaxation of a 2p electron into the 1s hole [K-shell fluorescence yield ω_K_ = 0.66; (*45*, *46*)], and emission of a 12.6-keV photon to conserve energy

$${\text{Kr}}^{+}(1s^{1*}\;2s^{2}\;2p^{6}\ldots 4p^{6})\to {\text{Kr}}^{+}(1s^{2}\;2s^{2}\;2p^{5*}\ldots 4p^{6})+P_{12.6\text{keV}}$$

The core-hole ion that is formed can also stabilize through Kβ emission and Auger emission. The detection of the fluorescent photons *P*_12.6keV_ is the goal of these experiments. The measured signal *I*_*x*,*y*_ collected from a position *x*,*y* in an axisymmetric 2D flow is related to the number of fluorescence photons *P*_12.6keV_ captured by the detector and can be given by

$$I_{x,y}=\eta_{d}(\frac{\Omega }{4\pi })(1-f_{\text{abs}})\omega ∆t∆x\;\pi {R_{P}}^{2}\sigma \phi n_{\text{Kr}(x,y)}$$ (1)

where η_d_ is the detector efficiency, Ω is the solid angle viewed by the detector, *f*_abs_ is the fraction of fluorescence photons directed toward the detector that are absorbed before reaching it, ω is the fluorescence yield, Δ*t* is the measurement time, Δ*x* is the detector field of view along the beam propagation direction, *R_P_* is the radius of the incident beam, σ is the absorption cross section, ϕ is the incident photon flux, and *n*_Kr(*x*,*y*)_ is the krypton number density. For a given experimental geometry and constant measurement time and assuming that the measurement is insensitive to compositional variations in the flame, the terms $\eta_{d}(\frac{\Omega }{4\pi })(1-f_{\text{abs}})\omega \text{∆}t\text{∆}x\;\pi {R_{P}}^{2}\sigma$ can be considered a calibration constant *c*, so Eq. 1 can be rewritten as

$$I_{x,y}=c\phi n_{\text{Kr}(x,y)}=c\phi X_{\text{Kr}(x,y)}{\left(\frac{N}{V}\right)}_{x,y}$$ (2)

where *X*_Kr(*x*,*y*)_ is the krypton mole fraction at position *x*,*y* in the flow and ${\left(\frac{N}{V}\right)}_{x,y}$ is the fluid density at position *x*,*y*. If the mole fraction of krypton and the pressure in the flow are constant, then Eq. 2 can be manipulated using the ideal gas law to obtain spatially resolved temperatures

$$I_{x.y}=c\phi X_{\text{Kr},\text{ref}}{\left(\frac{N}{V}\right)}_{x,y}=c\phi X_{\text{Kr},\text{ref}}\frac{P_{\text{ref}}}{T(x,y)k_{B}}$$ (3)

Furthermore, if a reference signal *I*_ref_ is collected at a place in the flow with known temperature *T*_ref_, the temperature can be further simplified as

$$T_{x,y}=\frac{I_{\text{ref}}}{I_{x,y}}T_{\text{ref}}$$ (4)

If the temperature remains constant rather than the krypton mole fraction, then the spatial krypton number densities can be calculated as

$$X_{\text{Kr}(x,y)}{\left(\frac{N}{V}\right)}_{x,y}=\frac{I_{x,y}}{I_{\text{ref}}}X_{\text{Kr},\text{ref}}{\left(\frac{N}{V}\right)}_{\text{ref}}=\frac{I_{x,y}}{I_{\text{ref}}}X_{\text{Kr},\text{ref}}(\frac{P_{\text{ref}}}{T_{\text{ref}}k_{b}})$$ (5)

where *I*_ref_ is a signal collected in a place with known mole fraction *X*_Kr,ref_ and constant pressure and temperature *P*_ref_ and *T*_ref_.

### Burner and flame details

In this study, an atmospheric pressure nonpremixed CH_4_ flame was generated with a Yale coflow burner (*28*, *47*). The burner geometry is shown in fig. S2. Flow rates and initial krypton mole fractions are shown in table S1. The krypton mole fractions were chosen to ensure sufficient signal while keeping the collection time reasonable. The scan settings are listed in table S2. For all experiments, the reactants either flowed from 99.99%+ purity cylinders (fuel-CH_4_, fuel-Kr, and oxidizer-Kr), an air compressor (oxidizer-air), or a house liquid nitrogen supply (fuel-N_2_). The flow rates of fuel-N_2_, fuel-CH_4,_ oxidizer-Kr, and oxidizer-air were controlled with FMA5400/5500 Omega thermal mass flow controllers that were directly calibrated with the corresponding process gas. The fuel-Kr flow rate was controlled with an MKS Series 1179A thermal mass flow controller. For the centerline measurements, a chimney with an internal diameter slightly larger than the coflow diameter was made of a Kapton sheet (5 mil) and placed atop the burner. The chimney was intended to minimize perturbations due to stray air currents.

For the atmospheric nonpremixed CH_4_ flame, Kr was seeded into both the fuel and oxidizer streams at the same initial mole fractions (<±1% variation). This requirement ensures that the mole fraction of Kr is constant throughout the flame, to within +2%/−3% (see fig. S4), so that the measured XRF signal is proportional to krypton number density. Using the ideal gas law, a temperature can then be computed from the measured Kr number densities. Uncertainties in measured temperatures due to phenomena such as nonuniform pressure, photon shot noise, or Kr mole fraction variations throughout the flame are discussed further on and are quantified in figs. S3 to S6.

### X-ray source and fluorescence detection

The experiments in this work were conducted at the 7-BM beamline of the APS at Argonne National Laboratory. The emission from the APS bending magnet x-ray source was filtered using a double multilayer monochromator, resulting in an x-ray beam at 15.0-keV nominal photon energy (Δ*E*/*E* = 1.0%). This beam was focused with a pair of Kirkpatrick-Baez focusing mirrors (260-mm optical length) to a focal spot 4 μm by 6 μm in size FWHM, with a beam divergence of less than 2 mrad in both directions. The burner was placed such that the center of the burner was at the same *z* (x-ray beam propagation direction) position as the focal point.

Three x-ray detectors were used in this work. A diamond photodiode (55-μm thickness) was used upstream of the focusing mirrors to monitor the incoming beam intensity. Downstream of the burner, a silicon PIN diode (300-μm thickness) was used to monitor the transmitted x-ray intensity. The signals from both photodiodes were amplified with transimpedance amplifiers and ported to voltage-to-frequency converters for readout. The x-ray emission spectra were recorded with a silicon drift diode (SDD) energy-dispersive x-ray detector (490-μm thickness). This detector was coupled to a digital x-ray pulse processor, providing a spectrum of the emitted x-rays. The detector was placed at 90° to the incident x-ray beam in the horizontal plane, taking advantage of the polarization of the x-ray beam to minimize the amount of x-ray scattering seen by the detector.

If used without other optics, the SDD would sense x-rays from a wide solid angle. To perform 2D-resolved fluorescence measurements, a polycapillary x-ray optic (100-mm focal length) was coupled to the detector in front of the SDD. The polycapillary effectively restricts x-rays from reaching the detector unless they originate from a small, well-defined region of the domain. The detector was positioned using a three-axis positioning platform, so the polycapillary focal region coincided with the x-ray beam focus. The polycapillary focal region was roughly Gaussian with a size of 270-μm FWHM at 15 keV. Accounting for the dependence of focal spot size on energy, the effective probe volume size of these measurements is 4 μm by 6 μm by 320 μm, with the largest dimension in the x-ray beam propagation direction.

To collect fluorescence data, the burner was moved using a precision *xy* positioning platform through a raster pattern. At each measurement location, the incident x-ray intensity, transmitted x-ray intensity, and x-ray emission were measured for 1 s. The 2D raster scan was used to build 2D maps of the flow field at various flow conditions.

Several steps were required to process these data. The incident and transmitted x-ray intensity were used to determine the attenuation of the beam in the flame. For each spectrum, a spectral region of interest containing the Kr Kα peak was defined. The Kr Kα photon counts were corrected for detector dead time effects, changes in incident beam intensity, and attenuation of the incident x-ray beam. Signal trapping effects were calculated to be quite minor, and hence, no correction for signal trapping was made.

### Flame simulations

The NGA code (*48*), an unsteady flow solver, was used to perform the 2D detailed simulations of the coflow diffusion flame. The scalar equations were discretized using the BQUICK scheme, which ensures that the physical bounds of appropriate quantities are numerically preserved throughout the simulation without adding substantial artificial diffusion (*49*). A recently developed computationally efficient, semi-implicit, iterative method is used for the time integration of chemical source terms for the transport equations of gas-phase species (*50*). The chemical model used in this work was constructed on the basis of the chemical model developed in (*51*, *52*). The original chemical model (*51*, *52*) contains 171 species and 1878 reactions (forward and backward reactions counted separately). The model has been extensively tested and validated in laminar diffusion flames (*53*–*55*); however, it does not contain Kr as inert gas. Therefore, the thermodynamic and transport properties of Kr were taken from (*56*, *57*) and (*57*), respectively, and were incorporated into the chemical model. For all simulations, a refined and uniform mesh was used around the burner exit where the main chemical reactions and diffusion processes occur. The mesh was gradually stretched in both radial and axial directions away from the burner exit to reduce the computational cost. The thermal boundary conditions for the fuel and oxidizer were taken as *T* = 298 K. The inlet flow conditions and burner geometry used for the current simulations are described in table S1 and fig. S1.

### Propagation of errors analysis

To estimate the uncertainty in the measured temperatures based on the synchrotron XRF technique, we used a propagation of errors analysis. The expression relating the measured fluorescent signal *I*_*x*,*y*_ to the local temperature *T*_*x*,*y*_ can be given as follows

$$T_{x,y}=c\phi X_{\text{Kr},\text{ref}}\frac{P_{\text{ref}}}{I_{x,y}k_{B}}$$ (6)

where *c* is the calibration constant, ϕ is the photon flux, *X*_Kr,ref_ is the assumed krypton mole fraction, *P*_ref_ is the assumed pressure, and *I*_*x*,*y*_ is the local fluorescent signal measured by the detector. The changes in *T*_*x*,*y*_ with respect to changes in *X*_Kr,ref_, *P*_ref_, and *I*_*x*,*y*_ are given by

$$\begin{array}{c} \frac{dT_{x,y}}{dX_{\text{Kr},\text{ref}}}=c\phi \frac{P_{\text{ref}}}{I_{x,y}k_{B}}\\ \frac{dT_{x,y}}{dI_{x,y}}=-c\phi X_{\text{Kr},\text{ref}}\frac{P_{\text{ref}}}{{I_{x,y}}^{2}k_{B}}\\ \frac{dT_{x,y}}{dP_{\text{ref}}}=c\phi \frac{X_{\text{Kr},\text{ref}}}{I_{x,y}k_{B}} \end{array}$$

Neglecting variations in *c* and ϕ, as well as covariances between the variables, the propagation of errors formula for estimating the standard deviation of *T*_*x*,*y*_ is

$$\sigma_{T}=\sqrt{{\frac{\mathrm{dT}}{\mathrm{dP}}}^{2}\sigma_{P}^{2}+{\frac{\mathrm{dT}}{dX_{\text{Kr},\text{ref}}}}^{2}\sigma_{X_{\text{Kr},\text{ref}}}^{2}+{\frac{\mathrm{dT}}{\mathrm{dI}}}^{2}\sigma_{S}^{2}}$$ (7)

where σ*_T_*, σ*_P_*, σ*_I_*, and σ_*X*_Kr, ref__ are the SDs in temperature, pressure, fluorescent signal, and krypton mole fraction. On the basis of mole fraction deviations presented in fig. S3, σ_*X*_Kr, ref__ can be estimated, based on two SDs, as 1.5%. The value for σ*_I_* can be determined from fig. S4. On the basis of the simulated 2D pressures in fig. S5, the overall pressure variations in the flame are less than 0.001%, and so σ*_P_* is negligible relative to other sources of uncertainty. Using Eq. 7, the spatial uncertainty in the flame temperatures can be computed, and is shown in fig. S6.

## References

[1] S. Galli, X-ray crystallography: One century of Nobel Prizes. J. Chem. Educ. 91, 2009–2012 (2014).

[2] K. Kohse-Höinghaus, Combustion in the future: The importance of chemistry. Proc. Combust. Inst. 38, 1–56 (2021). 10.1016/j.proci.2020.06.375 PMC7518234 33013234

[3] D. H. Bilderback, P. Elleaume, E. Weckert, Review of third and next generation synchrotron light sources. J. Phys. B: At., Mol. Opt. Phys. 38, S773–S797 (2005).

[4] C. A. MacDonald, Structured x-ray optics for laboratory-based materials analysis. Annu. Rev. Mat. Res. 47, 115–134 (2017).

[5] H. Sakurai, N. Kawahara, M. Itou, E. Tomita, K. Suzuki, Y. Sakurai, Densitometry and temperature measurement of combustion gas by x-ray Compton scattering. J. Synchrotron Radiat. 23, 617–621 (2016). 26917151 10.1107/S1600577516001740 PMC4804334

[6] N. Hansen, R. Tranter, J. Randazzo, J. Lockhart, A. Kastengren, Investigation of sampling-probe distorted temperature fields with x-ray fluorescence spectroscopy. Proc. Combust. Inst. 37, 1401–1408 (2019).

[7] N. Hansen, R. S. Tranter, K. Moshammer, J. B. Randazzo, J. P. A. Lockhart, P. G. Fugazzi, T. Tao, A. L. Kastengren, 2D-imaging of sampling-probe perturbations in laminar premixed flames using Kr x-ray fluorescence. Combust. Flame 181, 214–224 (2017).

[8] J. H. Frank, A. Shavorskiy, H. Bluhm, B. Coriton, E. Huang, D. L. Osborn, In situ soft x-ray absorption spectroscopy of flames. Appl. Phys. B 117, 493–499 (2014).

[9] A. L. Kastengren, C. F. Powell, T. Riedel, S.-K. Cheong, K.-S. Im, X. Liu, Y. J. Wang, J. Wang, Nozzle geometry and injection duration effects on diesel sprays measured by X-ray radiography. J. Fluids Eng. 130, 041301 (2008).

[10] E. Boigné, P. Muhunthan, D. Mohaddes, Q. Wang, S. Sobhani, W. Hinshaw, M. Ihme, X-ray computed tomography for flame-structure analysis of laminar premixed flames. Combust. Flame 200, 142–154 (2019). 30532316 10.1016/j.combustflame.2018.11.015 PMC6278941

[11] Y. A. Levendis, K. R. Estrada, H. C. Hottel, Development of multicolor pyrometers to monitor the transient response of burning carbonaceous particles. Rev. Sci. Instrum. 63, 3608–3622 (1992).

[12] GBD 2019 Risk Factors Collaborators, Global burden of 87 risk factors in 204 countries and territories, 1990–2019: A systematic analysis for the Global Burden of Disease Study 2019. The Lancet 396, 1223–1249 (2020). 10.1016/S0140-6736(20)30752-2 PMC7566194 33069327

[13] T. C. Bond, S. J. Doherty, D. W. Fahey, P. M. Forster, T. Berntsen, B. J. DeAngelo, M. G. Flanner, S. Ghan, B. Kärcher, D. Koch, S. Kinne, Y. Kondo, P. K. Quinn, M. C. Sarofim, M. G. Schultz, M. Schulz, C. Venkataraman, H. Zhang, S. Zhang, N. Bellouin, S. K. Guttikunda, P. K. Hopke, M. Z. Jacobson, J. W. Kaiser, Z. Klimont, U. Lohmann, J. P. Schwarz, D. Shindell, T. Storelvmo, S. G. Warren, C. S. Zender, Bounding the role of black carbon in the climate system: A scientific assessment. J. Geophys. Res. Atmos. 118, 5380–5552 (2013).

[14] N. J. Kempema, M. B. Long, Effect of soot self-absorption on color-ratio pyrometry in laminar coflow diffusion flames. Opt. Lett. 43, 1103–1106 (2018). 29489790 10.1364/OL.43.001103

[15] A. Sahoo, V. Narayanaswamy, Two-dimensional temperature field imaging in laminar sooting flames using a two-line Kr PLIF approach. Appl. Phys. B 125, 168 (2019).

[16] N. J. Kempema, M. B. Long, Quantitative Rayleigh thermometry for high background scattering applications with structured laser illumination planar imaging. Appl. Optics 53, 6688–6697 (2014). 10.1364/AO.53.006688 25322370

[17] J. Als-Nielsen, D. McMorrow, *Elements of Modern X-ray Physics* (John Wiley & Sons, 2011).

[18] A. Kastengren, C. F. Powell, Synchrotron X-ray techniques for fluid dynamics. Exp. Fluids 55, 1–15 (2014).

[19] G. Jauncey, The scattering of x-rays and Bragg’s law. Proc. Natl. Acad. Sci. U.S.A. 10, 57–60 (1924). 16576781 10.1073/pnas.10.2.57 PMC1085513

[20] J. Hubbell, S. Seltzer, Tables of X-ray mass attenuation coefficients and mass energy-absorption coefficients (version 1.4) (National Institute of Standards and Technology, 2004).

[21] K. Kohse-Höinghaus, R. S. Barlow, M. Aldén, J. Wolfrum, Combustion at the focus: Laser diagnostics and control. Proc. Combust. Inst. 30, 89–123 (2005).

[22] M. O. Krause, J. Oliver, Natural widths of atomic *K* and *L* levels, *K*α X-ray lines and several *KLL* Auger lines. J. Phys. Chem. Ref. Data 8, 329–338 (1979).

[23] C. K. Westbrook, Y. Mizobuchi, T. J. Poinsot, P. J. Smith, J. Warnatz, Computational combustion. Proc. Combust. Inst. 30, 125–157 (2005).

[24] M. Smooke, C. McEnally, L. Pfefferle, R. Hall, M. Colket, Computational and experimental study of soot formation in a coflow, laminar diffusion flame. Combust. Flame 117, 117–139 (1999).

[25] A. Kastengren, C. F. Powell, D. Arms, E. M. Dufresne, H. Gibson, J. Wang, The 7BM beamline at the APS: A facility for time-resolved fluid dynamics measurements. J. Synchrotron Radiat. 19, 654–657 (2012). 22713903 10.1107/S0909049512016883 PMC3579593

[26] R. S. Tranter, A. L. Kastengren, J. P. Porterfield, J. B. Randazzo, J. P. A. Lockhart, J. H. Baraban, G. B. Ellison, Measuring flow profiles in heated miniature reactors with X-ray fluorescence spectroscopy. Proc. Combust. Inst. 36, 4603–4610 (2017).

[27] International Sooting Flame (ISF) Workshop; https://www.adelaide.edu.au/cet/isfworkshop/

[28] M. J. Montgomery, D. D. Das, C. S. McEnally, L. D. Pfefferle, Analyzing the robustness of the yield sooting index as a measure of sooting tendency. Proc. Combust. Inst. 37, 911–918 (2019).

[29] N. J. Kempema, M. B. Long, Combined optical and TEM investigations for a detailed characterization of soot aggregate properties in a laminar coflow diffusion flame. Combust. Flame 164, 373–385 (2016).

[30] A. L. Bodor, B. Franzelli, T. Faravelli, A. Cuoci, A post processing technique to predict primary particle size of sooting flames based on a chemical discrete sectional model: Application to diluted coflow flames. Combust. Flame 208, 122–138 (2019).

[31] B. Franzelli, M. Roussillo, P. Scouflaire, J. Bonnety, R. Jalain, T. Dormieux, S. Candel, G. Legros, Multi-diagnostic soot measurements in a laminar diffusion flame to assess the ISF database consistency. Proc. Combust. Inst. 37, 1355–1363 (2019).

[32] M. L. Botero, N. Eaves, J. A. H. Dreyer, Y. Sheng, J. Akroyd, W. Yang, M. Kraft, Experimental and numerical study of the evolution of soot primary particles in a diffusion flame. Proc. Combust. Inst. 37, 2047–2055 (2019).

[33] D. Bartos, M. Sirignano, M. J. Dunn, A. D’Anna, A. R. Masri, Soot inception in laminar coflow diffusion flames. Combust. Flame 205, 180–192 (2019).

[34] J. S. Carlson, E. A. Monroe, R. Dhaoui, J. Zhu, C. S. McEnally, S. Shinde, L. D. Pfefferle, A. George, R. W. Davis, Biodiesel ethers: Fatty acid-derived alkyl ether fuels as improved bioblendstocks for mixing-controlled compression ignition engines. Energy Fuel 34, 12646–12653 (2020).

[35] H. Kwon, S. Lapointe, K. Zhang, S. W. Wagnon, W. J. Pitz, J. Zhu, C. S. McEnally, L. D. Pfefferle, Y. Xuan, Sooting tendencies of 20 bio-derived fuels for advanced spark-ignition engines. Fuel 276, 118059 (2020).

[36] B. D. Etz, G. M. Fioroni, R. A. Messerly, M. J. Rahimi, P. C. St. John, D. J. Robichaud, E. D. Christensen, B. P. Beekley, C. S. McEnally, L. D. Pfefferle, Y. Xuan, S. Vyas, R. S. Paton, R. L. McCormick, S. Kim, Elucidating the chemical pathways responsible for the sooting tendency of 1 and 2-phenylethanol. Proc. Combust. Inst. 38, 1327–1334 (2021).

[37] C. S. McEnally, Y. Xuan, P. C. St. John, D. D. Das, A. Jain, S. Kim, T. A. Kwan, L. K. Tan, J. Zhu, L. D. Pfefferle, Sooting tendencies of co-optima test gasolines and their surrogates. Proc. Combust. Inst. 37, 961–968 (2019).

[38] J. Bearden, X-ray wavelengths and X-ray atomic energy levels. NSRDS-NBS 14, 7 (1967).

[39] A. C. Eckbreth, *Laser diagnostics for combustion temperature and species* (CRC press, 1996), vol. 3.

[40] M. Smooke, M. Long, B. Connelly, M. Colket, R. Hall, Soot formation in laminar diffusion flames. Combust. Flame 143, 613–628 (2005).

[41] B. A. V. Bennett, C. S. McEnally, L. D. Pfefferle, M. D. Smooke, M. B. Colket, Computational and experimental study of axisymmetric coflow partially premixed ethylene/air flames. Combust. Flame 127, 2004–2022 (2001).

[42] N. J. Kempema, M. B. Long, Boundary condition thermometry using a thermographic-phosphor-coated thin filament. Appl. Optics 55, 4691–4698 (2016). 10.1364/AO.55.004691 27409027

[43] B. A. V. Bennett, C. S. McEnally, L. D. Pfefferle, M. D. Smooke, M. B. Colket, Computational and experimental study of the effects of adding dimethyl ether and ethanol to nonpremixed ethylene/air flames. Combust. Flame 156, 1289–1302 (2009).

[44] R. D. Deslattes, E.G. Kessler Jr., P. Indelicato, L. de Billy, E. Lindroth, J. Anton, J. S. Coursey, D. J. Schwab, J. Chang, R. Sukumar, K. Olsen, R. A. Dragoset, X-ray transition energies (version 1.2) (National Institute of Standards and Technology, 2005); [Online] http://physics.nist.gov/XrayTrans [accessed 20 October 2009].

[45] A. Kodre, M. Hribar, D. Glavič, The Auger-Raman effect and the K-shell fluorescence yield of krypton. Zeitschrift für Physik D Atoms, Molecules and Clusters 2, 173–176 (1986).

[46] V. O. Kostroun, M. H. Chen, B. Crasemann, Atomic radiation transition probabilities to the 1 s state and theoretical K-shell fluorescence yields. Phys. Rev. A 3, 533–545 (1971).

[47] J. Gau, D. Das, C. S. McEnally, D. Giassi, N. Kempema, M. Long, *Yale Coflow Burner Information and CAD Drawings*. Dataset (figshare, 2017); 10.6084/m9.figshare.5005007.v.

[48] O. Desjardins, G. Blanquart, G. Balarac, H. Pitsch, High order conservative finite difference scheme for variable density low Mach number turbulent flows. J. Comput. Phys. 227, 7125–7159 (2008).

[49] M. Herrmann, G. Blanquart, V. Raman, Flux corrected finite volume scheme for preserving scalar boundedness in reacting large-eddy simulations. AIAA J. 44, 2879–2886 (2006).

[50] B. Savard, Y. Xuan, B. Bobbitt, G. Blanquart, A computationally-efficient, semi-implicit, iterative method for the time-integration of reacting flows with stiff chemistry. J. Comput. Phys. 295, 740–769 (2015).

[51] G. Blanquart, P. Pepiot-Desjardins, H. Pitsch, Chemical mechanism for high temperature combustion of engine relevant fuels with emphasis on soot precursors. Combust. Flame 156, 588–607 (2009).

[52] K. Narayanaswamy, G. Blanquart, H. Pitsch, A consistent chemical mechanism for oxidation of substituted aromatic species. Combust. Flame 157, 1879–1898 (2010).

[53] G. Chen, W. Yu, J. Fu, J. Mo, Z. Huang, J. Yang, Z. Wang, H. Jin, F. Qi, Experimental and modeling study of the effects of adding oxygenated fuels to premixed n-heptane flames. Combust. Flame 159, 2324–2335 (2012).

[54] Y. Xuan, G. Blanquart, Numerical modeling of sooting tendencies in a laminar co-flow diffusion flame. Combust. Flame 160, 1657–1666 (2013).

[55] H. Kwon, A. Jain, C. S. McEnally, L. D. Pfefferle, Y. Xuan, Numerical investigation of the pressure-dependence of yield sooting indices for n-alkanes and aromatic species. Fuel 254, 115574 (2019).

[56] R. B. Bird, W. Stewart, E. Lightfoot, *Transport Phenomena, Revised 2nd Edition* (John Wiley & Sons Inc., 2006).

[57] E. Goos, A. Burcat, B. Ruscic, Extended third millennium ideal gas and condensed phase thermochemical database for combustion with updates from active thermochemical tables (German Aerospace Center, Institute of Combustion Technology, 2010) [accessed 2 August 2018].

[58] M. J. Montgomery, H. Kwon, J. A. H. Dreyer, Y. Xuan, C. S. McEnally, L. D. Pfefferle, Effect of ammonia addition on suppressing soot formation in methane co-flow diffusion flames. Proc. Combust. Inst. 38, 2497–2505 (2021).

