# Supplementary material for: In situ temperature measurements in sooting methane/air flames using synchrotron x-ray fluorescence of seeded krypton atoms
Source: Sci Adv. 2022 Apr 29;8(17):eabm7947. doi: 10.1126/sciadv.abm7947 (PMC9054011; doi:10.1126/sciadv.abm7947)
Supplement: Supplementary file 1 — Figs. S1 to S6 Tables S1 and S2 [file sciadv.abm7947_sm.pdf]

Science Advances
AAAS

Supplementary Materials for

In situ temperature measurements in sooting methane/air flames using synchrotron x-ray fluorescence of seeded krypton atoms

Matthew J. Montgomery*, Hyunguk Kwon, Alan L. Kastengren, Lisa D. Pfefferle, Travis Sikes, Robert S. Tranter, Yuan Xuan, Charles S. McEnally

*Corresponding author. Email: matthew.montgomery@yale.edu

Published 29 April 2022, *Sci. Adv.* **8**, eabm7947 (2022)
DOI: 10.1126/sciadv.abm7947

This PDF file includes:

Figs. S1 to S6
Tables S1 and S2

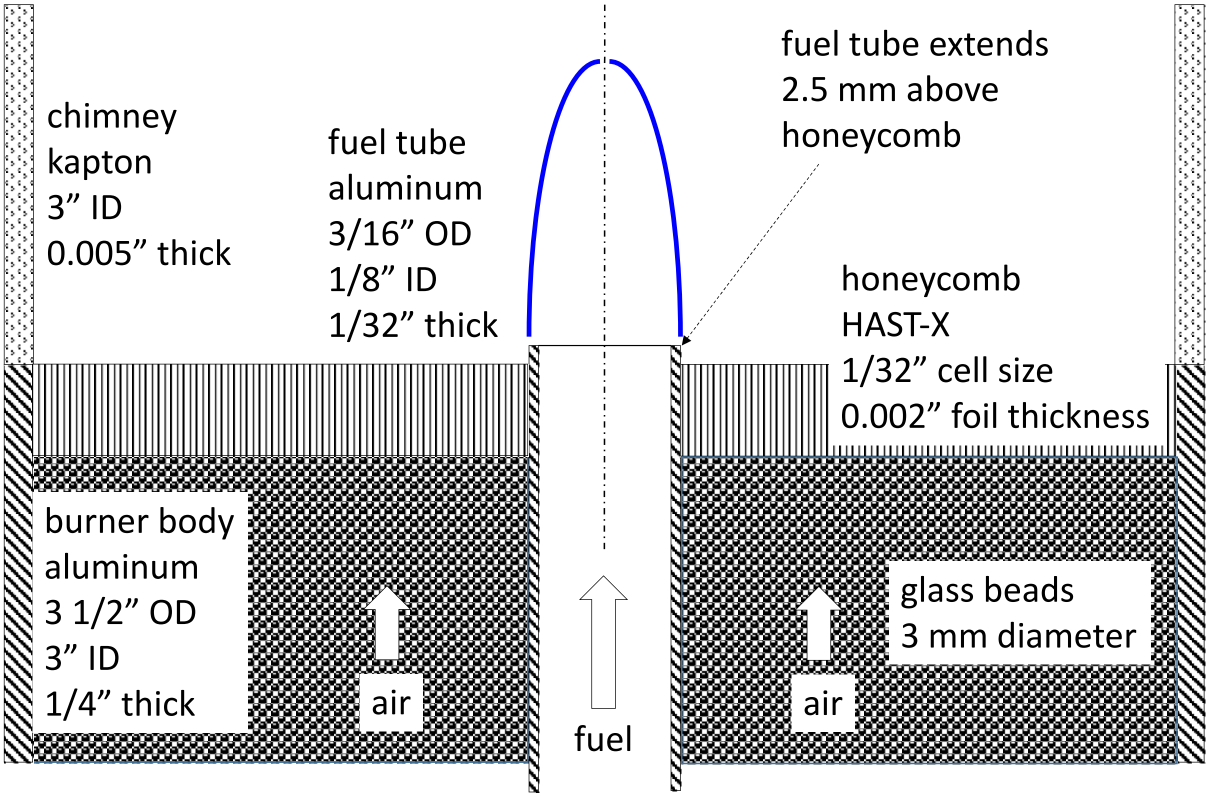

Fig. S1. Diagram of the burner with specific dimensions.

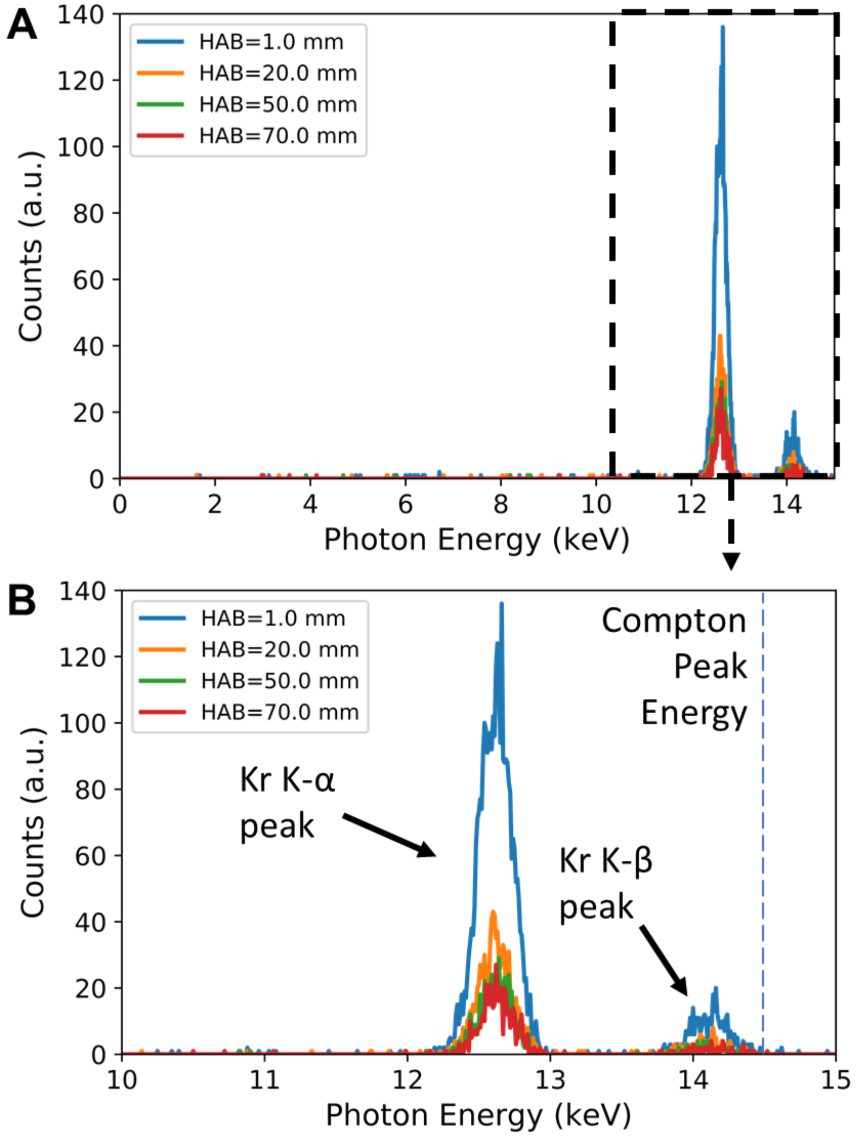

Fig. S2. Measured detector signal as a function of photon energy, at various HABs along the centerline of the flame. The incident photon energy was 15 keV. (A) Detector signal across the entire spectrum from 0 to 15 keV. (B) Zoomed-in region of the spectrum from 10 to 15 keV, where the fluorescence signal was detected.

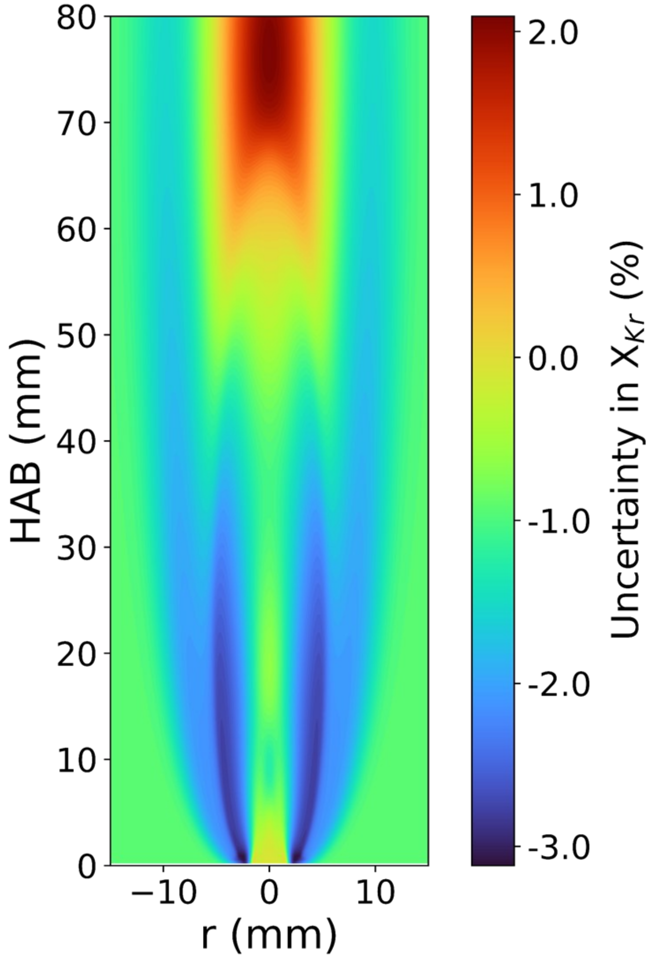

Fig. S3. Error in the measurements due to krypton mole fraction variations. The uncertainty was calculated based on the simulated krypton mole fractions and the assumed experimental value listed in Table S1.

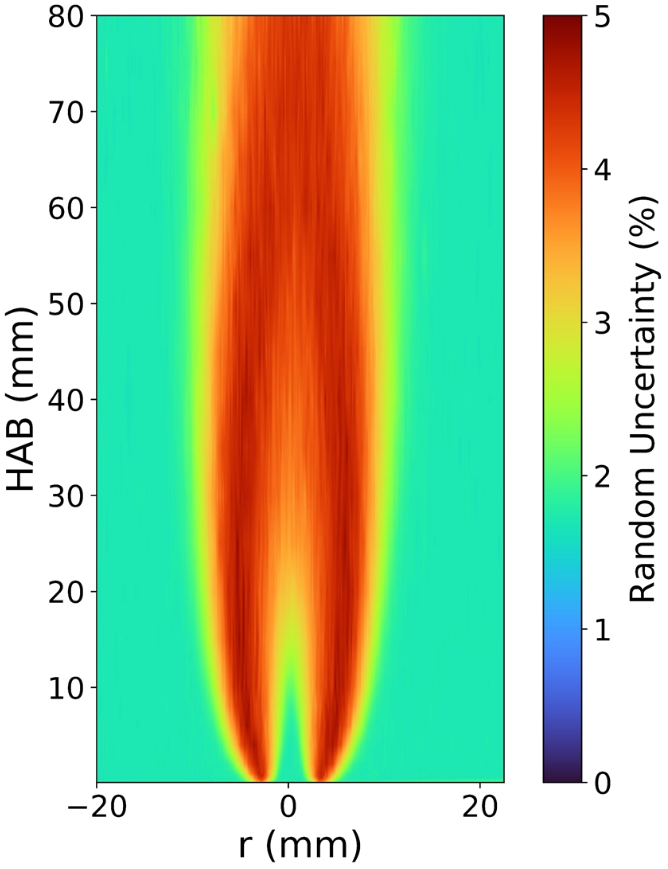

Fig. S4. Random uncertainty in the measurements attributable to photon shot noise. Uncertainties are shown for one standard deviation.

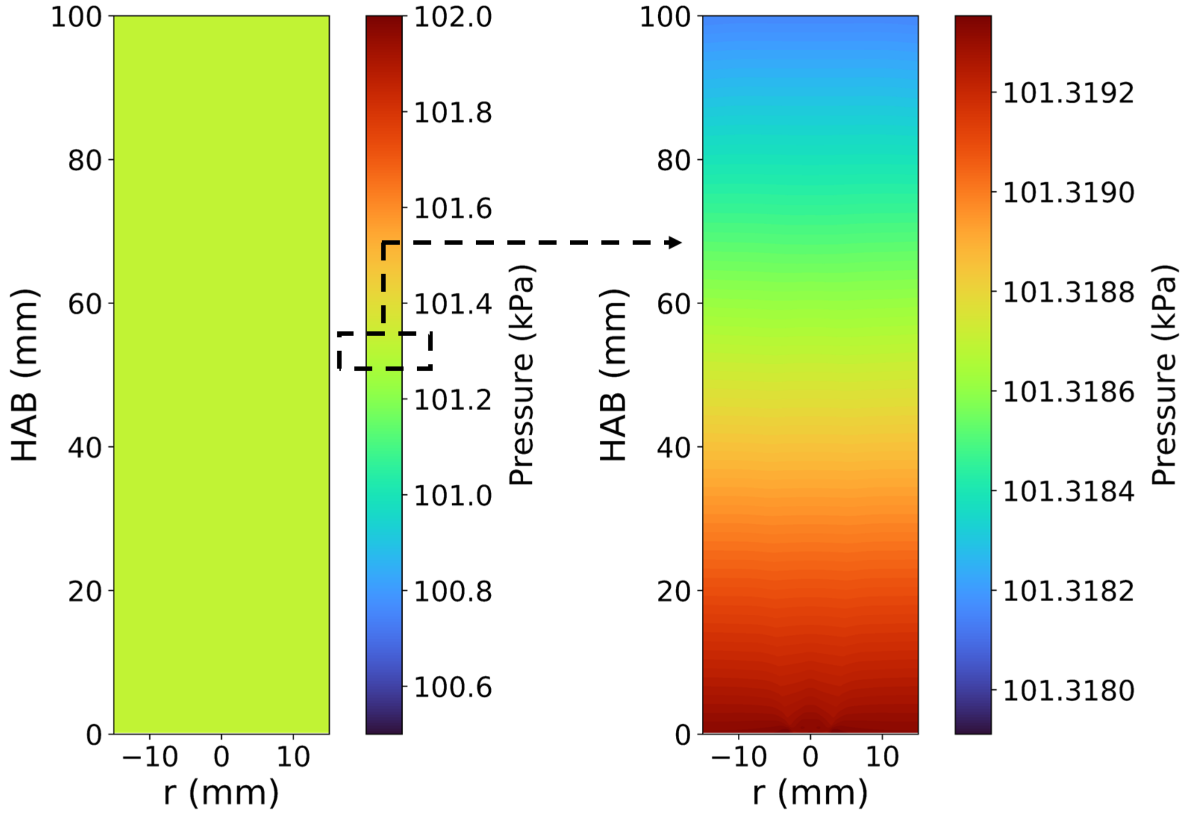

Fig. S5. Simulated 2D Pressures in the Nonpremixed CH_4 Flame Seeded with Krypton. The maximum uncertainty from pressure-variations is <0.01%, and is considered negligible relative to other sources of uncertainty.

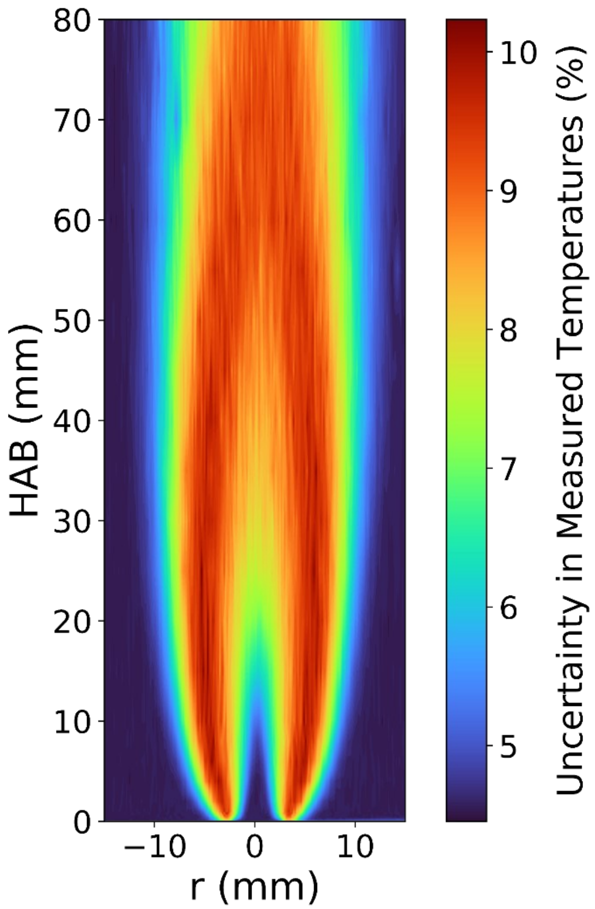

Fig. S6. Spatial uncertainty in measured 2D temperatures. Uncertainties are calculated according to the propagation of errors analysis presented above, and are based on two standard deviations.

**Atmospheric nonpremixed CH_4/air flame**						
**Fuel**				**Oxidizer**		
**N_2 (mL/min)**	**CH_4 (mL/min)**	**Kr (mL/min)**	**$X_{Kr,fuel}$**	**Air (L/min)**	**Kr (mL/min)**	**$X_{Kr,ox}$**
0	330	8.0	0.0237	50.0	1206.9	0.0236

Table S1. Experimental flow rates for the sooting methane/air co-flow flame analyzed in this study

**Configuration**	**Type of Scan/Collection Time**	**Raster Scan Settings**	
Atmospheric Nonpremixed CH_4-air flame	2-D (Figures 1/2/4)<br>Collection time: 1 s<br>Total time: 2 hrs	**HAB**<br>0.1 to 0.2mm: 0.1 mm/step<br>0.2 to 1 mm: 0.2 mm/step<br>2 to 10 mm: 2 mm/step<br>10 to 80 mm: 5mm/step<br>**25 steps total**	**Radial position**<br>-20 to +22.5 mm: 0.1496 mm/step<br>**284 steps total**
	1-D (Figure 2C)<br>Collection time: 20 s<br>Total time: 42 min	**HAB**<br>0.4 to 2 mm: 0.2 mm/step<br>2 to 20 mm: 0.5 mm/step<br>20 to 100 mm: 1 mm/step<br>**125 steps total**	**Radial position**<br>r=0 mm (centerline)

Table S2. Raster scan settings used in this study.
